# The importance of assessing vision in disabled children – and how to do it

**Published:** 2016

**Authors:** Richard Bowman

**Affiliations:** Senior Lecturer: Public Health Ophthalmology, London School of Hygiene and Tropical Medicine, London, UK.

**Figure:**
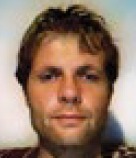
Richard Bowman

Children with disabilities are at a much higher risk of having visual impairment than those without disabilities (10.5% vs 0.16%)^1^ and are also at higher risk of other ocular disorders such as strabismus and refractive error. One reason for this higher rate among disabled children is that brain injury (such as pre-natal asphyxia or prematurity) underlies a range of disabilities, including learning disabilities and sensory impairments.

Sadly, however, children with disabilities often have poorer access to eye services (see page 9), even though it is possible for their visual function and eye health to be accurately assessed – as we will show in this article.

## Clinical history taking

It is important to take a careful history from either the child (depending on age, development and communication ability), or from the main carer of the child, or both. Carers can be asked if they have noticed whether the child has any problems moving around or finding things, or responding to a smile – both of which can be related to poor vision. Children with blindsight (see panel opposite), but who are not mobile, may rock to and fro. They do this to create apparent motion of their visual environment in order to obtain some visual stimulation.

You may wish to adapt how you respond to children depending on the type of impairment that the child has.

- **Child with intellectual impairments.** It is useful to know the developmental age of the child so that language and questions can be developmental age appropriate. When talking to the child it may be helpful to slow your voice down and to exaggerate facial expressions.
- **Child with hearing impairments.** Mild hearing impairment may require slower, louder speech, making sure the child can see your mouth (as they may also lip read). The consultation should be in a quiet room. More severe hearing impairment may require sign language.
- **Child with mobility impairments.** It is important to make the clinic comfortable and easy for these children to access. It may be necessary to make some changes to the clinic, such as a ramp for wheelchairs or for children who use crutches. Alternatively, children might have to be assessed at home. Handheld equipment such as a portable slit lamp may be needed.
- **Child with behavioural difficulties.** Assessing these children requires patience, a quiet environment and finding out from carers when is the best time of the day for an assessment.

**Figure:**
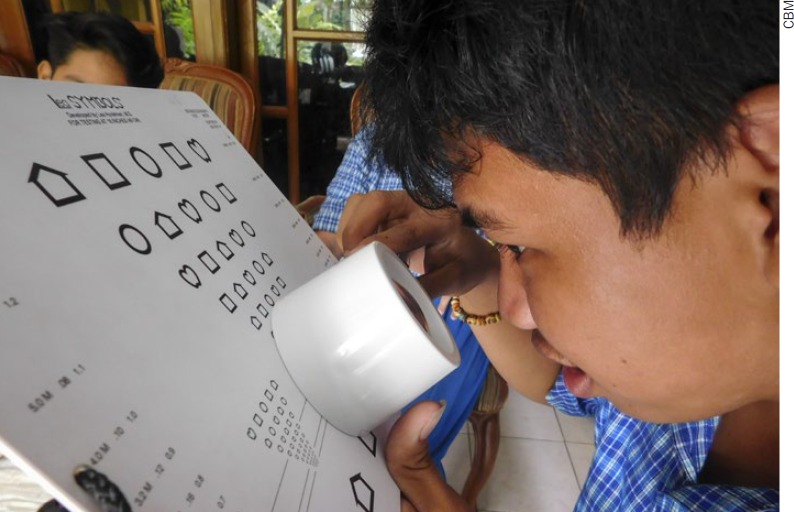
A child with a hearing impairment undergoes a low vision assessment at the school for children with disabilities which he attends. CAMBODIA

Gordon Dutton and his team at the Royal Hospital for Sick Children, Glasgow have developed the Visual Skills Inventory, which can be a useful basis for taking a structured history.^2^ For children with profound motor and/or intellectual impairments, the shorter version of this inventory (the Short Visual Skills Questionnaire^3^, adapted by the Vision Science Research Group at Ulster University) is useful. It contains questions such as:

- Does she know and recognise your face? Other peoples' faces?
- Does he react to you approaching him?
- Does she react to a light being switched on?
- Does he screw up his eyes in bright light?
- Does she return your silent smile?
- Does he reach for objects? What size?
- Is she aware of a spoonful of food coming towards her mouth? From both sides?
- Does his vision seem better in bright or dim light?

For children with less profound disability and better vision it is often possible to take a more detailed history, looking for evidence of visual-perceptual problems.

For these patients, the full Visual Skills Inventory^3^ is helpful. It is longer with 53 questions and is useful for older and less profoundly disabled children. It has been shown to be reliable and has been used effectively in the UK^4^ and in Bangladesh^5^ to elicit problems with higher visual function in disabled children.

The inventory can be particularly helpful for children with a mild disability (motor or intellectual), and often with good visual acuity, who seem to have a lot of trouble visually processing the real, busy, crowded, moving world. Their problems can include difficulty finding objects or recognising faces, difficulty walking on uneven ground, and difficulty seeing moving objects.

Once their problems are better understood, suggestions can be made on how the impact can be minimised to improve the quality of life of these children and their carers. The Visual Skills Questionnaire now has specific recommendations^3^ for adapting the environment and behaviour of the child both at home and at school, depending on the answers given. The family or carers will therefore have a set of recommendations which are tailored to the particular needs of their child.

For example, if a child cannot recognise faces, the parent can agree to wear clothes of a particular colour when meeting them outside school. If there is loss of vision in the lower field and poor visual control of the legs, then a child is taught to stop and look down before negotiating rough ground or Steps. Such simple changes can be surprisingly effective.

In addition to all of the above, it is important to take a routine medical history in order to understand the cause of the child's disability. For example, a child born prematurely and who has cerebral palsy is likely to have cerebral visual impairment and/or visual-perceptual problems. A child without disabilities whose parents report that her development has stopped, or even gone backwards (i.e. she can no longer read), may have a rare neuro-metabolic condition with retinal manifestations, e.g. Batten's disease.

## Clinical examination

To assess a disabled child, we should ideally be in a calm environment, when the child is not too tired. Vision can fluctuate significantly according to environment and tiredness, especially for children with cerebral visual impairment (see panel). It is important that you, the child, and the parents or carers are feeling relaxed, so allow enough time for the examination and schedule it when the child is likely to be most active and alert.

The examination should be thorough and cover all the basics, as disabled children are more likely – not less likely – to have abnormal visual findings. Research into children with cerebral palsy in Bangladesh and India showed that the majority could perform preferential looking visual acuities e.g. Cardiff Cards or Teller compared to recognition tests such as Snellen or Lea recognition/matching.

Preferential looking tests are detection tests, not recognition tests, and the results should not be taken as equivalent to Snellen values even if the preferential looking card has a ‘Snellen equivalent’ written on it. Nevertheless, they are very useful for pre-verbal or non-verbal children. The choice of visual acuity charts should be decided by the child's mental, not chronological age. For example, a 10-year old child with an IQ of 50 would respond well to a visual acuity chart that measures the vision of a 4- or 5-year old.

Visual fields can be assessed by confrontation. Field defects are quite common in disabled children, particularly if there is brain pathology. Evidence of a hemianopia or bilateral lower visual field loss (which is common) can be obtained by basic confrontation techniques. Functional vision can be assessed briefly using the questions from the Insight visual skills inventory as a basis, especially if the carer is not sure of the answers to these questions.

Eye movements should be assessed using standard methods. Slit lamp examination can be performed for a child in a wheelchair using a side-on approach or sometimes by removing the foot platforms. Portable slit lamps, such as those used for babies, can also be useful for very immobile children.

**Figure:**
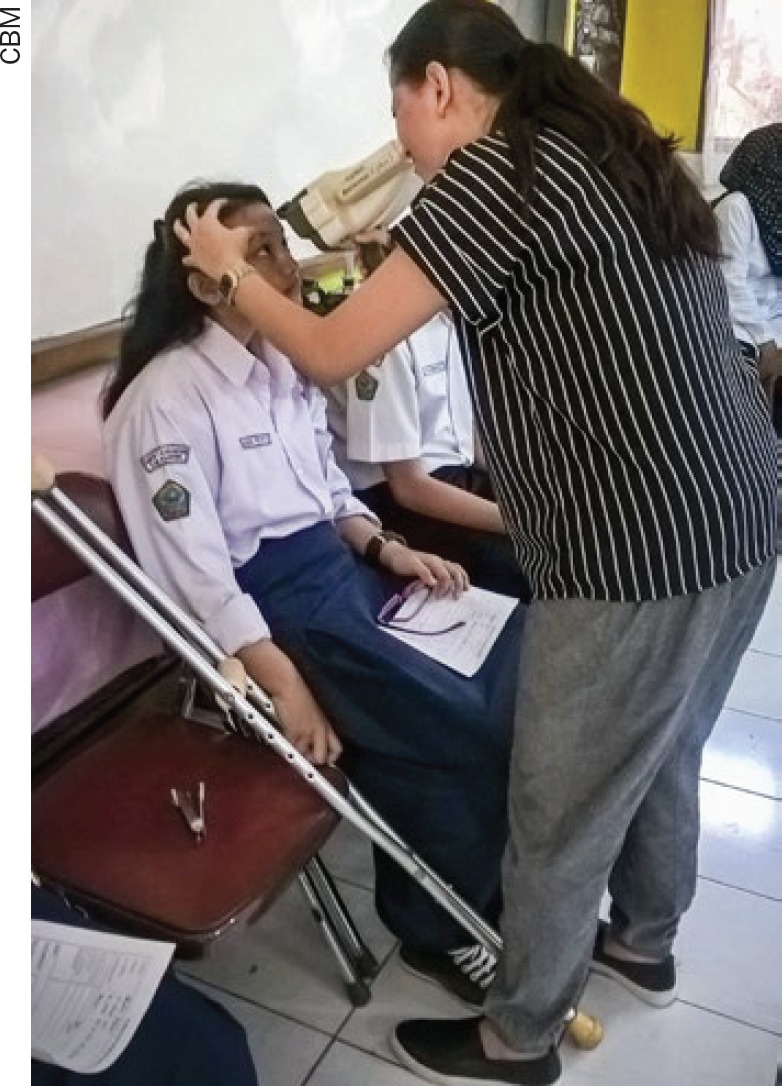
Portable equipment makes eye examinations easier to carry out

Dilated fundoscopy, at least once, is essential (as for any paediatric examination). Ask yourself:

- Are the optic discs swollen, which might suggest hydrocephalus?
- Are the optic discs pale or smaller than usual?
- Is there cataract in children with Down's syndrome?
- Is there retinal pathology, which can offer a hint about the underlying condition?

Finally, cycloplegic refraction is one of the most important parts of the examination. There is a high rate of refractive error in children with disabilities and spectacles may be the safest and most effective intervention we can give. Even if they are emmetropic, many children have poor accommodation and therefore are relatively impaired for near vision. It may well be worth a trial of +3.00 spectacles, even at an early age (when the main visual world of interest is near). These spectacles also give magnification when there is reduced acuity.

The lives of children with disabilities are significantly improved when they receive the eye care they need, Children with disabilities are at greater risk of having visual impairment and eye care professionals should therefore actively seek out and provide eye care to disabled children – it is worth the effort.

**Vision and the brain**

The function of the eyes is to generate clear, focused images which are transmitted to the visual cortex of the brain via the optic nerves and optic tracts.

From the visual cortex, information is transmitted to multiple other areas of the brain (higher centres) via ventral and dorsal pathways, so that the visual information can be interpreted (‘I know that face’) or acted upon (a faster heart beat after seeing a snake on the path in front of you). Some of the reactions are conscious, but many are subconscious, for example ‘blindsight’. This is the ability of some people with profound visual impairment to see movement which allows them to navigate reasonably well without bumping into things.

Many children who are disabled have problems with **seeing** (due to damage in the pathways leading to the visual cortex). Other disabled children have difficulties **interpreting** visual information as a result of damage to the pathways leading **from the visual cortex** to the higher centres, or asa result of damage to the higher centres themselves; these are called visual-perceptual problems.

**Figure 1.**
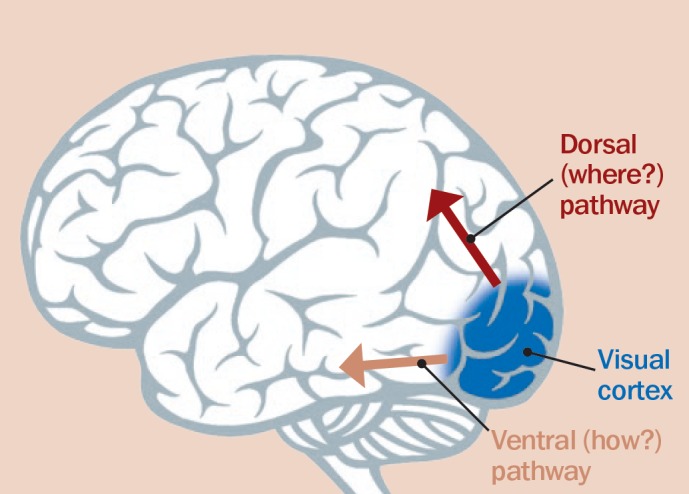
Diagram of the visual pathways
